# Electrooxidation Using Nb/BDD as Post-Treatment of a Reverse Osmosis Concentrate in the Petrochemical Industry

**DOI:** 10.3390/ijerph16050816

**Published:** 2019-03-06

**Authors:** Salatiel Wohlmuth da Silva, Carla Denize Venzke, Júlia Bitencourt Welter, Daniela Eduarda Schneider, Jane Zoppas Ferreira, Marco Antônio Siqueira Rodrigues, Andréa Moura Bernardes

**Affiliations:** 1 Universidade Federal do Rio Grande do Sul (UFRGS), Instituto de Pesquisas Hidráulicas (IPH), Av. Bento Gonçalves, Porto Alegre/RS 9500, Brazil; 2 Universidade Federal do Rio Grande do Sul (UFRGS), Programa de Pós-Graduação em Engenharia de Minas, Metalúrgica e de Materiais (PPGEM), Av. Bento Gonçalves, Porto Alegre/RS 9500, Brazil; carladenize@gmail.com (C.D.V.); juliabwelter@gmail.com (J.B.W.); danielaeduardaschneider@hotmail.com (D.E.S.); jane.zoppas@ufrgs.br (J.Z.F.); amb@ufrgs.br (A.M.B.); 3 Universidade Feevale, Campus II ERS-239, Novo Hamburgo 2755, RS, Brazil; marcor@feevale.br

**Keywords:** petrochemical wastewater, reverse osmosis concentrate, boron-doped diamond on niobium substrate, electrochemical advanced oxidation

## Abstract

This work evaluated the performance of an electrochemical oxidation process (EOP), using boron-doped diamond on niobium substrate (Nb/BDD), for the treatment of a reverse osmosis concentrate (ROC) produced from a petrochemical wastewater. The effects of applied current density (5, 10, or 20 mA·cm^−2^) and oxidation time (0 to 5 h) were evaluated following changes in chemical oxygen demand (COD) and total organic carbon (TOC). Current efficiency and specific energy consumption were also evaluated. Besides, the organic byproducts generated by EOP were analyzed by gas chromatography coupled to mass spectrometry (GC–MS). The results show that current densities and oxidation time lead to a COD and TOC reduction. For the 20 mA·cm^−2^, changes in the kinetic regime were found at 3 h and associated to the oxidation of inorganic ions by chlorinated species. After 3 h, the oxidants act in the organic oxidation, leading to a TOC removal of 71%. Although, due to the evolution of parallel reactions (O_2_, H_2_O_2,_ and O_3_), the specific energy consumption also increased, the resulting consumption value of 66.5 kW·h·kg^−1^ of COD is considered a low energy requirement representing lower treatment costs. These results encourage the applicability of EOP equipped with Nb/BDD as a treatment process for the ROC.

## 1. Introduction

The large volume of water used in industrial processes, in association with water scarcity, leads the industries to develop practices of economy and reuse of process water [1]. Commonly, industries apply physico-chemical and biological processes to treat the wastewater. However, these processes are not capable to generate water of the standard required for reuse. There are several types of water reuse. Considering that in the petrochemical industry the highest water consumption occurs in cooling towers, the reuse water should not contain ions and organic matter, which can lead to corrosion and incrustation [2].

Petrochemical wastewater is an environmental concern, showing high toxicity and recalcitrant compounds, such as phenolic compounds or aromatic amines. In addition, it can contain large amounts of hydrocarbons, metal derivatives, surface-active substances, sulfides, naphthenic acids, aniline, nitrobenzene, organochlorines, leading to a high chemical oxygen demand concentration and low biodegradability [3,4,5]. If it is released in the environment without the proper treatment, it poses risks to human health and the ecosystem [6].

Membrane technologies have been applied for desalination and wastewater treatment [7]. Reverse osmosis (RO) uses a pressurized membrane module for desalination and advanced treatment of different wastewater matrices. As final products, the process generates a high-quality water and a concentrate [8]. The high-quality water can be reused in industrial processes. However, the challenge of RO is the management of the generated concentrate. The untreated or improperly managed concentrate can result in adverse environmental effects, due to high concentration of inorganic and organic contaminants, including contaminants of emerging concern [9]. For this reason, it is important to develop efficient concentrated treatment techniques, able to achieve water in a quality respecting environmental regulations [4].

Integrated treatment systems are one of the strategies for improving the quality and minimizing the quantity of concentrate. The combined systems could involve hybrid processes, RO and advanced oxidation processes (AOPs) such as RO/Photo-assisted electrochemical [10], RO/Fenton [10], RO/UV radiation in the C spectrum (UV-C)/H_2_O_2_ [11], RO/Electrochemical oxidation with Cu–graphite [12] and RO/Electrochemical oxidation with boron-doped diamond on silicon substrate (Si/BDD) [13]. Electrochemical oxidation processes (EOP) are an interesting option to treat the RO concentrate, obtained from petrochemical wastewater, and the use of the boron-doped diamond on niobium substrate (Nb/BDD) anode in RO/EOP system is still a matter or research. The Nb/BDD was chosen instead of the classical Si/BDD, because Nb/BDD can save energy, when compared to Si/BDD, due to the high conductivity nature of Nb [14].

In this study, EOP was evaluated using the Nb/BDD anode aiming for the reduction of the organic contaminant load from the RO concentrate (ROC). The treatment train is composed of pretreatment/RO/EOP equipped with the Nb/BDD anode. Anodic current densities of 5, 10 and 20 mA·cm^−2^ were applied to ensure an anode potential that allows hydroxyl radicals (${\mathrm{HO}}^{•}$) generation (>2.1 V vs. Ag/AgCl). The performance of EOP was evaluated based on the chemical oxygen demand reduction and mineralization of the organic load present in the ROC. Current efficiency and specific energy consumption was also estimated. Besides, gas chromatography mass spectroscopy (GC–MS) was used to monitor the formation of byproducts at the end of the treatment time.

## 2. Materials and Methods

### 2.1. Wastewater

The petrochemical industry situated, in South Brazil, has a conventional wastewater treatment plant (CWWTP) composed of a physico-chemical treatment (primary), an extended-aeration modified activated sludge (secondary) and eight stabilization ponds (tertiary). The wastewater used in the RO experiments was collected after the tertiary wastewater treatment at the end of the eighth pond. To remove the largest particles and to avoid damage in the RO system, the collected wastewater was subjected to a pretreatment with sand filters (SF) and activated carbon (AC) as described in a previous publication [1].

### 2.2. Reverse Osmosis and Electrochemical Advanced Oxidation Setup

**Figure 1:**
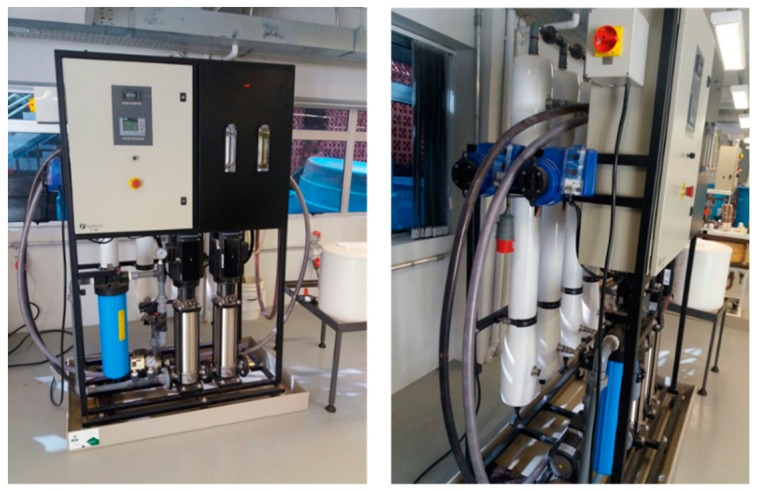
Reverse osmosis (RO) pilot system (PAM Selective Membranes, Rio de Janeiro, Brazil) equipped by a spiral polyamide membrane module BW 30 (4040) (Filmtec, Dow Chemical, Midland, MI, USA) with a membrane area of 7.2 m^2^.

The assays were carried out in a semi-continuous process. Firstly, the RO assays were performed in a pilot system from PAM Selective Membranes, Rio de Janeiro, Brazil, equipped with a spiral polyamide membrane module BW 30 4040 (Filmtec), with a membrane area of 7.2 m^2^ (Figure 1). Based on the best-operating conditions, determined in the previous work of Venzke et al. [15], the RO unit was operated with a transmembrane pressure (∆P) of 8 bar, while the permeate flow was maintained at 348 L·h^−1^ and the reject flow at 300 L·h^−1^. The low pressure used is associated with the low osmotic pressure of the wastewater to be treated.

Under these conditions the conductivity of the permeate was kept constant with a low specific energy consumption. The tests were carried out in batch with a volume of 10,000 L. All assays were performed in triplicate at 25 °C.

After the wastewater treatment by the RO system, an ROC volume of 1.5 L was placed on a double-jacket reservoir that feeds the electrochemical cell in recirculation mode through a peristaltic pump at a flow rate of 180 L·h^−1^. The temperature was regulated at 25 °C by circulating external thermostated water/ethylene glycol (2:1) between the inner and outer reservoir walls.

A one-compartment flow reactor with capacity of 0.3 L was used for all assays. The anode was a boron-doped diamond (BDD) thin film, obtained by hot filament chemical vapor deposition (CVD) technique, on niobium (Nb) substrate, purchased from NeoCoat SA (Switzerland). A monolayer polycrystalline film with thickness of 2.5 ± 0.2 µm, 5000 ppm of boron concentration, and a diamond-sp^3^/sp^2^-carbon ratio of ~200 was obtained. The cathode was a stainless steel AISI 304L. The geometric area of all electrodes in contact with the ROC was 0.01 m^2^. The EOP was carried out in galvanostatic mode by applying a constant current of 5, 10, or 20 mA·cm^−2^. When these current densities were applied in the EOP, the cell potential did no change with time, meaning that no electrode poisoning or passivation had occurred (Tables S1–S3 in Supplementary Materials).

**Figure 2:**
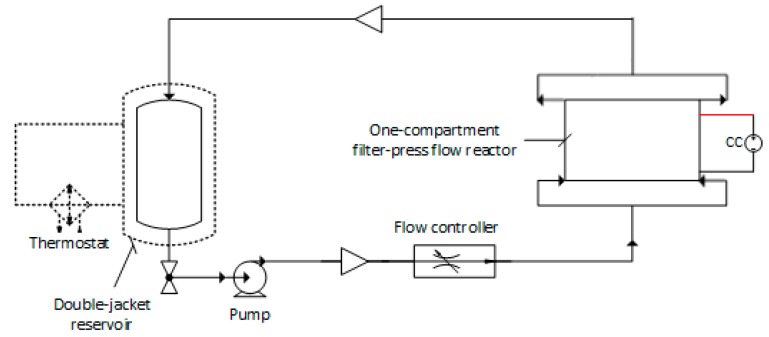
Flowchart of the electrochemical oxidation process (EOP) for organic removal from reverse osmosis concentrate (ROC). The red line is the anode contact. The dashed is the circulating external thermostated liquid (water/ethylene glycol).

All EOP were in triplicate, and samples were collected at time 0 and every 1-h until a final treatment time of 5 h. All experiments were conducted at an initial pH of 8 (the original ROC). A scheme of the experimental set-up used for ROC treatment by EOP with a Nb/BDD cell is presented in Figure 2.

According to the literature, the EOP process can be operated in two different regimes, under current or mass transport control, depending on the applied current density (i), the concentration of chemical oxygen demand (COD, mol O_2_ m^−3^), the oxidation time, and the value of the limiting current density (i_lim_) [16].

When i ≤ i_lim_, the EOP is under current control, the COD decreases linearity with time and the current efficiency should be 100%. On the other hand, if i ≥ i_lim_, the EOP is under mass transport control and the COD decay follows an exponential pattern with time. In this way, the current efficiency tends to decrease due to the increase of parasitic reactions, such as the oxygen evolution reaction (OER).

According to Equation (1), the i_lim_ can be estimated.

(1) $$i_{\lim}=4\cdot F\cdot k_{m}.\mathrm{COD}$$

where $i_{\lim}$ is the limiting current density (A·m^−2^), F is the Faraday constant (C·mol^−1^), $k_{m}$ is the mass transport coefficient (m·s^−1^), and COD is the chemical oxygen demand at the beginning of the EOP.

Taking into account Equation (1), and the initial value of COD considered as 1.25 mol O_2_ m^−3^, the mass transport coefficient is estimated as 4.97 × 10^−6^ m·s^−1^, by using the pair $\left(\mathrm{Fe}{\left(\mathrm{CN}\right)}_{6}^{4-}\right)$/$\left(\mathrm{Fe}{\left(\mathrm{CN}\right)}_{6}^{3-}\right)$ [17], the i_lim_ of the EOP system is calculated resulting in 0.24 mA·cm^−2^. It means that for all applied current densities used in this work (5, 10, and 20 mA·cm^−2^), the process will be under mass transport control.

To estimate the accumulative current efficiency (ϕ, %) and the specific energy consumption (Es, kW·h·kg^−1^) the Equations (2) and (3) were employed [18].

(2) $$\phi =\frac{F\;\times \;V\;\times \;\Delta {\left(\mathrm{COD}\right)}_{\exp}}{8\times \int_{0}^{t}I\left(t\right)\mathrm{dt}}$$

where F is the Faraday constant (C·mol^−1^), V is the volume of ROC (L) placed on the double-jacket reservoir, $\Delta {\left(\mathrm{COD}\right)}_{\exp}$ is the experimental COD decay (g L^−1^), 8 is the oxygen equivalent mass (g·eq^−1^), and I is the applied current.

(3) $$E_{s}=\frac{\int_{0}^{t}U\left(t\right)\times I\left(t\right)\mathrm{dt}}{1000\times V\times \Delta {\left(\mathrm{COD}\right)}_{\exp}}$$

where U is the cell voltage (V) and 1000 is the factor to homogenize units (W to kW and mg to kg).

### 2.3. Analysis

Alkalinity, color, chemical oxygen demand (COD), hardness, ammoniacal nitrogen, total Kjeldahl nitrogen (TKN), total dissolved solids, total suspended solids, total solids, fecal coliforms, and total coliforms were analyzed following the Standard Methods for the Examination of Water and Wastewater [19]. Chlorides, phosphorus, nitrate, nitrite, sodium, and sulfate were analyzed by ion chromatography (ICS 5000, Dionex/Thermo Fisher Scientific, Waltham, MA, USA). Calcium and total iron were determined by a flame atomic absorption spectrometer (FAAS) in a Varian SpectrAA110 system (Varian, Palo Alto, CA, USA). Conductivity and pH were monitored (Multiparameter 900, Bante instruments, Shanghai, China). Turbidity was measured by an Alfakit turbidimeter (Alfakit, Florianópolis, Brazil).

Total organic carbon (TOC), total carbon (TC), and inorganic carbon (IC) were determined on a Shimadzu TOC-LCPH equipped with an automatic sample injector (Kyoto, Japan). All procedures were accomplished according to the Shimadzu standard manual. To determine the quantity of organically bound carbon, the organic molecules must be broken down and converted to a single molecular form that can be measured quantitatively. The TOC method utilized at this work uses high temperature (680 °C) and regular catalysts (spheres coated with platinum) to convert organic carbon to carbon dioxide (CO_2_) (Equation (4)). The produced CO_2_ was purged from the sample, dried, and transferred with a carrier gas (oxygen 6.0) to a nondispersive infrared analyzer (Shimadzu, Kyoto, Japan).

(4) $$C_{x}H_{y}O_{z}\to {\mathrm{CO}}_{2}+H_{2}O$$

For ROC and final samples treated by EOP with Nb/BDD anode, qualitative analyses were performed by gas chromatography coupled to mass spectroscopy (GC–MS, LECO Corporation, St. Joseph, MI, USA). Comprehensive Two-Dimensional Gas Chromatography with Time-of-Flight Mass Spectrometer (GCxGC-TOFMS, LECO 7890A, LECO Corporation, St. Joseph, MI, USA) equipped with an automatic injector was used to analyze the samples. The qualitative analyses were done in SCAN mode between 35 and 400 m/z by GC–MS. The column was a low-polarity capillary column Rxi-5 SiMS 30 m × 0.25 mm × 0.25 μm (Restek, Evry, France). The mass spectrophotometer was operated in electron impact mode at 70 eV. The flow rate of the carrier gas was 1 mL min^−1^ and the injector was set at 250 °C. The oven temperature program started at 60 °C for 1 min, then a ramp profile increase of 18 °C·min^−1^ was applied up to 200 °C, and, after a hold for 6 min, 6 °C·min^−1^ was applied up to 280 °C when there was another hold for 15 min. The injected volume was 1 μL. The injection was carried out in pulsed Split mode. The transfer line was set at 250 °C. The detected products were qualitative identified by comparison with the NISTMS-2008 library.

## 3. Results and Discussion

### 3.1. Reverse Osmosis Concentrate (ROC)

It is possible to see in Table 1 that the RO permeate presented a quality for water reuse in the cooling towers (CT), which means that this technology is suitable for producing water for industrial reuse [20,21]. However, the concentrate from the RO process has organic compounds that can have a serious impact on water bodies if improperly discharged. For this concern, EOP using Nb/BDD anode could be an option to remove the organic load before discarding into water bodies.

### 3.2. Removal of Organic Content from ROC by EOP

**Figure 3:**
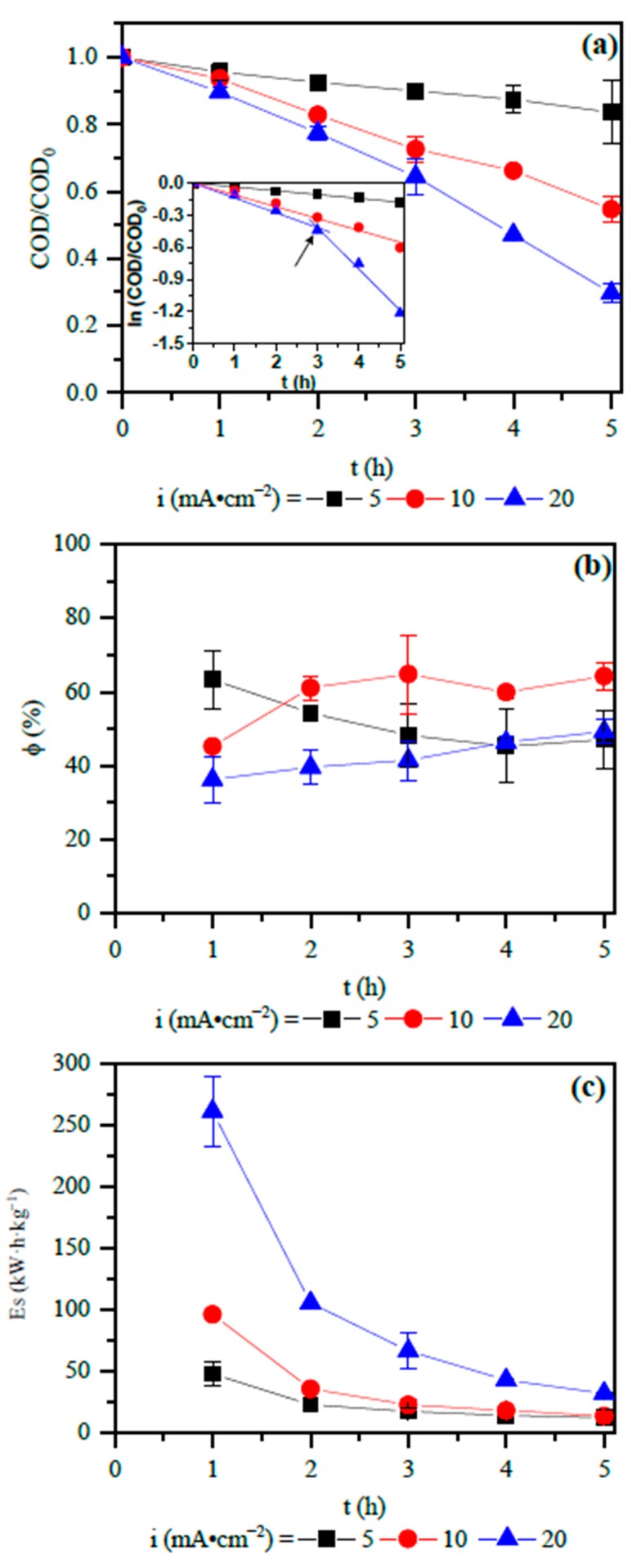
Influence of applied current densities in: (**a**) normalized chemical oxygen demand (COD) decay of ROC treated by EOP by using a Nb/BDD anode. The insert graphic is the kinetic modelling; (**b**) current efficiency and (**c**) specific energy consumption.

The effect of applied current densities of 5, 10, and 20 mA·cm^−2^ on ROC oxidation was studied and is illustrated in Figure 3a. As reported in the literature [22], higher oxidation is expected for higher applied currents, and this effect was observed in Figure 3a. For the applied current densities of 5, 10, and 20 mA·cm^−2^ the COD reductions at the end of 5 h were 17%, 48%, and 75%, respectively. TOC removal is considered to be the mineralization of the organic carbon (Equation (4)) and presented a similar result to the COD (14%, 44%, and 75%, respectively, see Figure S1 Supplementary Materials).

Figure 3a also shows a linear decay of COD for all applied current densities. Nevertheless, an exponential COD decay was expected, since the mass transfer controls EOP in this work. As commented, the limiting current of the system used in this work was estimated in 0.24 mA·cm^−2^ and the current densities used were 5, 10, and 20 mA·cm^−2^. The linear COD decay by a mass transfer controlled EOP was also found in the literature [23], and can be explained by the presence of ions (see Table 1) on the ROC. These ions lead to competitive reactions at the anode surface, between the oxidation of the organic compounds (R) present in the ROC (Equations (4) and (5)) and the generation of oxidant species (Equations (6)–(11)) and the oxidation of ${\mathrm{NO}}_{X}$ compounds by these oxidant species.

To reinforce the result that EOP is under mass transport limitations, it is noted in Figure 3b that the current efficiency was much smaller than 100% [16]. However, the identified values are typical for electrochemical processes [24].

(5) $$\mathrm{Nb}/\mathrm{BDD}+H_{2}O\to \mathrm{BDD}\;\left({\mathrm{HO}}^{•}\right)+H^{+}+e^{-}$$

(6) $$\mathrm{Nb}/\mathrm{BDD}\;\left({\mathrm{HO}}^{•}\right)+R\to {\;\mathrm{CO}}_{2}+H_{2}O+\mathrm{inorganic}\;\mathrm{ions}$$

(7) $${\mathrm{Cl}}^{-}+{\mathrm{HO}}^{•}\to {\mathrm{ClO}}^{-}+H^{+}+e^{-}$$

(8) $${\mathrm{ClO}}^{-}+{\mathrm{HO}}^{•}\to {\mathrm{ClO}}_{2}^{-}+H^{+}+e^{-}$$

(9) $${\mathrm{ClO}}_{2}^{-}+{\mathrm{HO}}^{•}\to {\mathrm{ClO}}_{3}^{-}+H^{+}+e^{-}$$

(10) $${\mathrm{ClO}}_{3}^{-}+{\mathrm{HO}}^{•}\to {\mathrm{ClO}}_{4}^{-}+H^{+}+e^{-}$$

(11) $${\mathrm{HSO}}_{4}^{-}+{\mathrm{HO}}^{•}\to {\mathrm{SO}}_{4}^{-•}+H_{2}$$

(12) $${\mathrm{SO}}_{4}^{-•}+{\mathrm{SO}}_{4}^{-•}\to S_{2}O_{8}^{2-}$$

A first-order kinetics reaction modelling expressed by the Equation (13), can represent the COD decay:

(13) $$\ln\left(\frac{\mathrm{COD}}{{\mathrm{COD}}_{0}}\right)=-k^{\prime }\times t$$

where ${\mathrm{COD}}_{0}$ is the COD value (g O_2_ L^−1^) at the initial treatment time (t = 0), COD represents the COD at n time, and $k^{\prime }$ is the rate constant (h^−1^) for oxidation of ROC.

The insert graphic in Figure 3a shows the shape of the natural logarithm (ln) of COD/COD_0_ as a function of the treatment time. For an applied current density between 5 and 10 mA·cm^−2^ the ln follows a linear pattern, and a good linear straight line was obtained with R^2^ > 0.989. From the slope, the k′ of 0.0355 h^−1^ and 0.1102 h^−1^ was found for 5 and 10 mA·cm^−2^, respectively. The first-order abatement is typical of the removal of organic compounds by EOP [24] and can be explained by the quasi-steady content achieved for ${\mathrm{HO}}^{•}$ and with the typical mass transfer coefficients (about 10^−5^ m·s^−1^). The ${\mathrm{HO}}^{•}$ radicals cannot accumulate on the anode surface or in the bulk solution because they have a very short lifetime [25]. From 0 h to 3 h of oxidation, a similar pattern of COD reduction was found when 20 mA·cm^−2^ was applied (k′ = 0.1384 h^−1^). However, after 3 h of treatment time, there is a higher increase in the kinetic constant, resulting in the value of 0.3900 h^−1^.

As mentioned, the EOP was conducted at pH 8 and low COD concentration. Under these conditions there may be a predominance of oxidant species based on chlorine leading first to indirect oxidation of ${\mathrm{NO}}_{x}$ compounds, not favoring COD reduction [26]. An increment in current density from 10 to 20 mA·cm^−2^ may further increase the production of chlorinated and sulfate oxidant species, instead of hydroxyl radicals, on the anode surface, favoring ${\mathrm{NO}}_{x}$ oxidation for the first 3 h of treatment time. After that, all oxidants generated act in the indirect oxidation of the organic content in the ROC, increasing the kinetic constant of COD reduction [27]. The current efficiency and the specific energy consumption also corroborated this finding. Indeed, the current efficiency rises after 3 h of EOP when 20 mA·cm^−2^ was applied (Figure 3b), also diminishing the specific energy consumption (Figure 3c).

The current efficiency and specific energy consumption depend on the operating conditions as applied current density, dissolved oxygen, pH, temperature, and inorganic ions, as well as the anode material properties [24]. In fact, the only parameter that can be directly controlled is the applied current density. As explained previously in the removal of COD from ROC by EOP, different applied currents favor the formation of other oxidants like chlorine, ozone, hydrogen peroxide, persulfate, and sulfate radicals [28]. These oxidants favor the maximization of parasitic oxygen evolution reactions and the oxidation of inorganic instead of organic compounds, leading to an increase in the specific energy consumption.

### 3.3. Detection of Organic Compounds from ROC and Byproducts after EOP

All products detected by GC–MS and shown in the chromatograms were identified by comparison with the NISTMS-2008 library (more details in Table 2).

In the ROC (Table 2 and Figure S2 in Supplementary Materials), 2-pyrrolidone, benzoic acid, and 1-docosene were found. 2-pyrrolidone is used as a non-corrosive high-boiling polar solvent and as an intermediate in the production of polymers such as polyvinylpyrrolidone and polypyrrolidone. Benzoic acid is mainly used in the production of glycol benzoates for the application of plasticizer in adhesive formulations. 1-docosene is used as lubricant, additive and viscosity adjustor. With these considerations, it is possible to find these compounds in the petrochemical wastewater.

After the treatment by EOP there is a change in the byproducts identified. When 5 mA·cm^−2^ was applied, part of the benzoic acid was probably converted into the identified product caprolactam (Table 2 and Figure S2 in Supplementary Materials) [29]. As the applied current density increased to 10 mA·cm^−2^ (Table 2 and Figure S2 in Supplementary Materials, probably all the benzoic acid was converted to caprolactam. However, when 20 mA·cm^−2^ (Table 2 and Figure S2 in Supplementary Materials) was applied in the EOP, caprolactam was not identified, and presumably the benzoic acid was oxidized to CO_2_ and H_2_O.

For all applied currents in the EOP, 1,6-dioxacyclododecan-7,12-dione was found, that is probably a degradation product of dibutyl phthalate. Besides that, for an applied current of 5 or 10 mA·cm^−2^, 1-docosene was not identified as a byproduct. In fact, as shown previously (Equations (4–11)), distinct applied currents can lead to different oxidant species, as a consequence, differences in the degradation pathway can be found.

It is noted that when chlorine is present, undesired byproducts could be produced. However, the production of undesired chlorinated byproducts was not detected in this work. This finding can be linked to the fact that the EOP was conducted at pH 8 (Figure S3 in Supplementary Materials) and, according to the literature, chlorinated organic compounds and gaseous chlorine may be formed only at around pH 3 [30].

## 4. Conclusions

This study showed that the EOP can be an alternative treatment for organic removal from the ROC. At the best operating conditions (20 mA·cm^−2^ and 5 h of treatment), a mineralization of 71%, current efficiency of 42 ± 4 and specific energy consumption of 66.5 ± 1 were found, showing satisfactory results. The oxidation was carried out mainly by indirect oxidation by hydroxyl radicals, chlorinated and sulfated oxidant species produced on the anode surface. For the best operating condition, a change in the kinetic regime was found and attributed to the preferential oxidation of inorganic ions in the first 3 h of treatment.

The lower energy requirements represent lower treatment costs. Finally, the present work encourages the applicability of EOP equipped with Nb/BDD as a treatment process for the reduction of the organic load present in the ROC.

## Figures and Tables

**Table 1:** Water quality of pre-and-post reverse osmosis (RO) treatment.

Parameters	RO Feed	RO Permeate	RO Concentrate	CT *	Units	Methodology
Alkalinity	85.9	3.6	132.3	n.s.	mg CaCO_3_·L^−1^	Titrimetry
Total Organic Carbon (TOC)	16.65	n.d.	25.42	1300	mg C·L^−1^	TOC
Chlorides	85.9	6.4	129.5	500	mg·L^−1^	IC
Conductivity	1255	18.30	1879.50	n.s.	μS·cm^−1^	Conductivimetry
Color	21	3	30	n.s.	Pt-Co	Colorimetry
Chemical Oxygen Demand (COD)	21.5	n.d.	39.4	300	mg O_2_·L^−1^	Colorimetry
Hardness	115.1	n.d.	181.6	n.s.	mg CaCO_3_·L^−1^	Titrimetry
Phosphorus	1.551	0.058	2.169	n.s.	mg L^−1^	IC
Nitrate	n.d.	0.1813	0.1045	n.s.	mg·L^−1^ N-NO_3_	IC
Nitrite	0.003	n.d.	0.005	n.s.	mg·L^−1^ N-NO_2_	IC
Ammoniacal nitrogen	n.d.	n.d.	n.d.	n.s.	mg·L^−1^	Titrimetry
Total nitrogen Kjeldahl	n.d.	n.d.	n.d.	n.s.	mg·L^−1^	Titrimetry
pH	7.37	6.12	7.38	n.s.	-	Potentiometry
Total Dissolved Solids	746	n.d.	1231	n.s.	mg·L^−1^	Gravimetry
Total Suspended Solids	n.d.	n.d.	2.3	100	mg·L^−1^	Gravimetry
Total solids	748	6.0	1248	n.s.	mg·L^−1^	Gravimetry
Sulfates	1110	10.92	86,959	n.s.	mg·L^−1^	IC
Turbidity	0.9	0.3	1.2	n.s.	NTU	Turbidimetry
Fecal Coliforms	Not present	Not present	Not present	10^4^	MPN·100 mL^−1^	Enzyme Substrate
Total Coliforms	9.3 × 10^1^	Not present	1.3 × 10^1^	10^4^	MPN·100 mL^−1^	Enzyme Substrate
Calcium	27.96	0.352	49.385	n.s.	mg·L^−1^	FAAS
Total iron	n.d.	n.d.	n.d.	n.s.	mg·L^−1^	FAAS
Sodium	144	5.60	218	n.s.	mg·L^−1^	IC

n.d.—Not detected by the method. n.s—Non-specified. NTU—Nephelometric turbidity unit. MPN—Most probable number. * Water quality for using on cooling tower.

**Table 2:** Compounds identified by tentative comparison with the NISTMS-2008 library with more than 90% of probability in ROC and EOP applying 5, 10, or 20 mA·cm^−2^.

Retention Time (min)	Compounds Identification	Molecular Formula	Average Mass (Da)	Similarity (%)
ROC				
5.02	2-Pyrrolidone	C_4_H_7_NO	85.104	94
6.14	Benzoic acid	C_7_H_6_O_2_	122.121	91
18.84	1-Docosene	C_22_H_44_	308.585	96
EOP—5 mA·cm^−2^				
5.02	2-Pyrrolidone	C_4_H_7_NO	85.104	94
6.14	Benzoic acid	C_7_H_6_O_2_	122.121	91
7.00	Caprolactam	C_6_H_11_NO	113.1576	93
9.22	1,6-Dioxacyclododecan-7,12-dione	C_10_H_16_O_4_	200.232	94
EOP—10 mA·cm^−2^				
5.02	2-Pyrrolidone	C_4_H_7_NO	85.104	95
6.97	Caprolactam	C_6_H_11_NO	113.1576	90
9.20	1,6-Dioxacyclododecan-7,12-dione	C_10_H_16_O_4_	200.232	95
13.83	Dibutyl phthalate	C_6_H_22_O_4_	278.344	93
EOP—20 mA·cm^−2^				
4.99	2-Pyrrolidone	C_4_H_7_NO	85.104	94
9.19	1,6-dioxacyclododecane-7,12-dione	C_10_H_16_O_4_	200.232	92
13.80	Dibutyl phthalate	C_6_H_22_O_4_	278.344	94
18.79	1-Docosene	C_22_H_44_	308.585	96

## Supplementary Materials

The following are available online at https://www.mdpi.com/1660-4601/16/5/816/s1, Table S1: Applied current density of 5 mA·cm^−2^ and the achieved cell potential with time, Table S2: Applied current density of 10 mA·cm^−2^ and the achieved cell potential with time, Table S3: Applied current density of 20 mA·cm^−2^ and the achieved cell potential with time, Figure S1: Influence of applied current densities on the (a) TOC removal and (b) COD reduction, Figure S2. Chromatograms for (a) Reverse Osmosis Concentrate (ROC) before the application of EOP and after the EOP applying (b) 5 mA·cm^−2^, (c) 20 mA·cm^−2^ and (d) 30 mA·cm^−2^.
