# Supplementary material for: Electrooxidation Using Nb/BDD as Post-Treatment of a Reverse Osmosis Concentrate in the Petrochemical Industry
Source: Int J Environ Res Public Health. 2019 Mar 6;16(5):816. doi: 10.3390/ijerph16050816 (PMC6427655; doi:10.3390/ijerph16050816)
Supplement: Supplementary file 1 [file ijerph-16-00816-s001.pdf]

International Journal of
*Environmental Research and Public Health*

MDPI

# Electrooxidation using Nb/BDD as post-treatment of a reverse osmosis concentrate in a petrochemical industry

**Salatiel Wohlmuth da Silva[1,2*], Carla Denize Venzke[2], Júlia Bitencourt Welter[2], Daniela Eduarda Schneider[2], Jane Zoppas Ferreira[2], Marco Antônio Siqueira Rodrigues[3], Andréa Moura Bernardes[2]**

1 Universidade Federal do Rio Grande do Sul (UFRGS) – Instituto de Pesquisas Hidráulicas (IPH). Av. Bento Gonçalves, 9500, Porto Alegre/RS, Brasil; salatiel.silva@ufrgs.br

2 Universidade Federal do Rio Grande do Sul (UFRGS) - Programa de Pós-Graduação em Engenharia de Minas, Metalúrgica e de Materiais (PPGEM). Av. Bento Gonçalves, 9500, Porto Alegre/RS, Brasil; carladenize@gmail.com; juliabwelter@gmail.com; danielaeduardaschneider@hotmail.com; jane.zoppas@ufrgs.br; amb@ufrgs.br

[2] Universidade Feevale, Campus II ERS-239, 2755, Novo Hamburgo, RS, Brasil.; marcor@feevale.br

* Correspondence: salatiel.silva@ufrgs.br; Phone number: +55 51 3308 9430; Av. Bento Gonçalves, 9500 - Setor IV - Prédio 43426 - Sala 103, Porto Alegre, Rio Grande do Sul, Brasil

**Table S1.** Applied current density of 5 mA·cm$^{-2}$ and the achieved cell potential with time.

| Time (h) | Cell potential (V) |
|---|---|
| 0 | 12.3 |
| 1 | 12.4 |
| 2 | 12.3 |
| 3 | 12.3 |
| 4 | 12.3 |
| 5 | 12.3 |

**Table S2.** Applied current density of 10 mA·cm$^{-2}$ and the achieved cell potential with time.

| Time (h) | Cell potential (V) |
|---|---|
| 0 | 22.8 |
| 1 | 22.5 |
| 2 | 22.5 |
| 3 | 22.8 |
| 4 | 22.4 |
| 5 | 22.1 |

**Table S3.** Applied current density of 20 mA·cm$^{-2}$ and the achieved cell potential with time.

| Time (h) | Cell potential (V) |
|---|---|
| 0 | 38.5 |
| 1 | 38.7 |
| 2 | 38.5 |
| 3 | 38.5 |
| 4 | 38.5 |
| 5 | 38.5 |

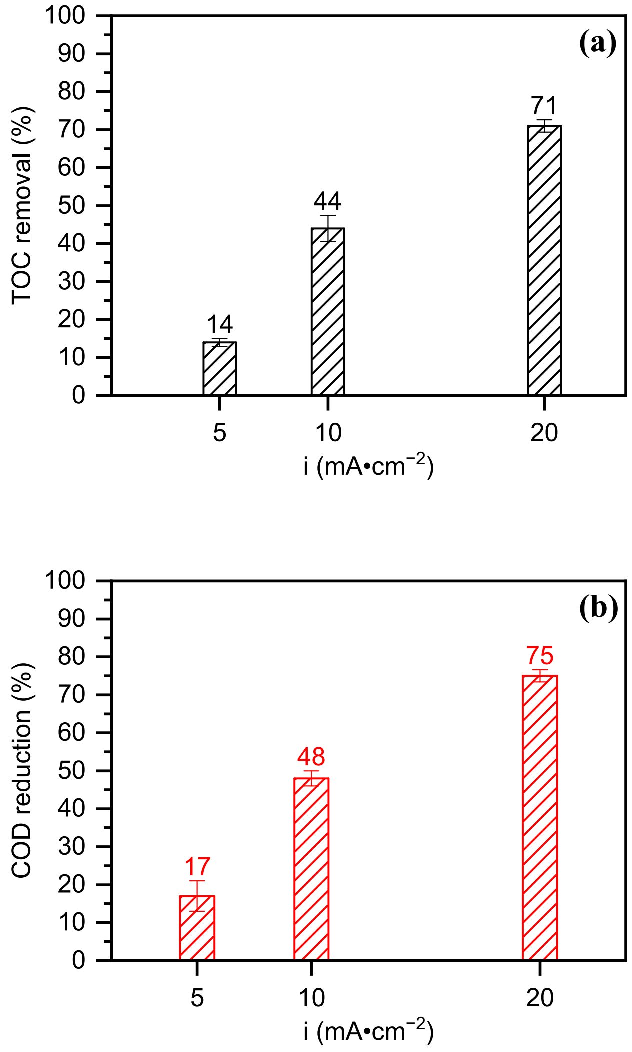


**Figure S1.** Influence of applied current densities on the **(a)** TOC removal and **(b)** COD reduction.

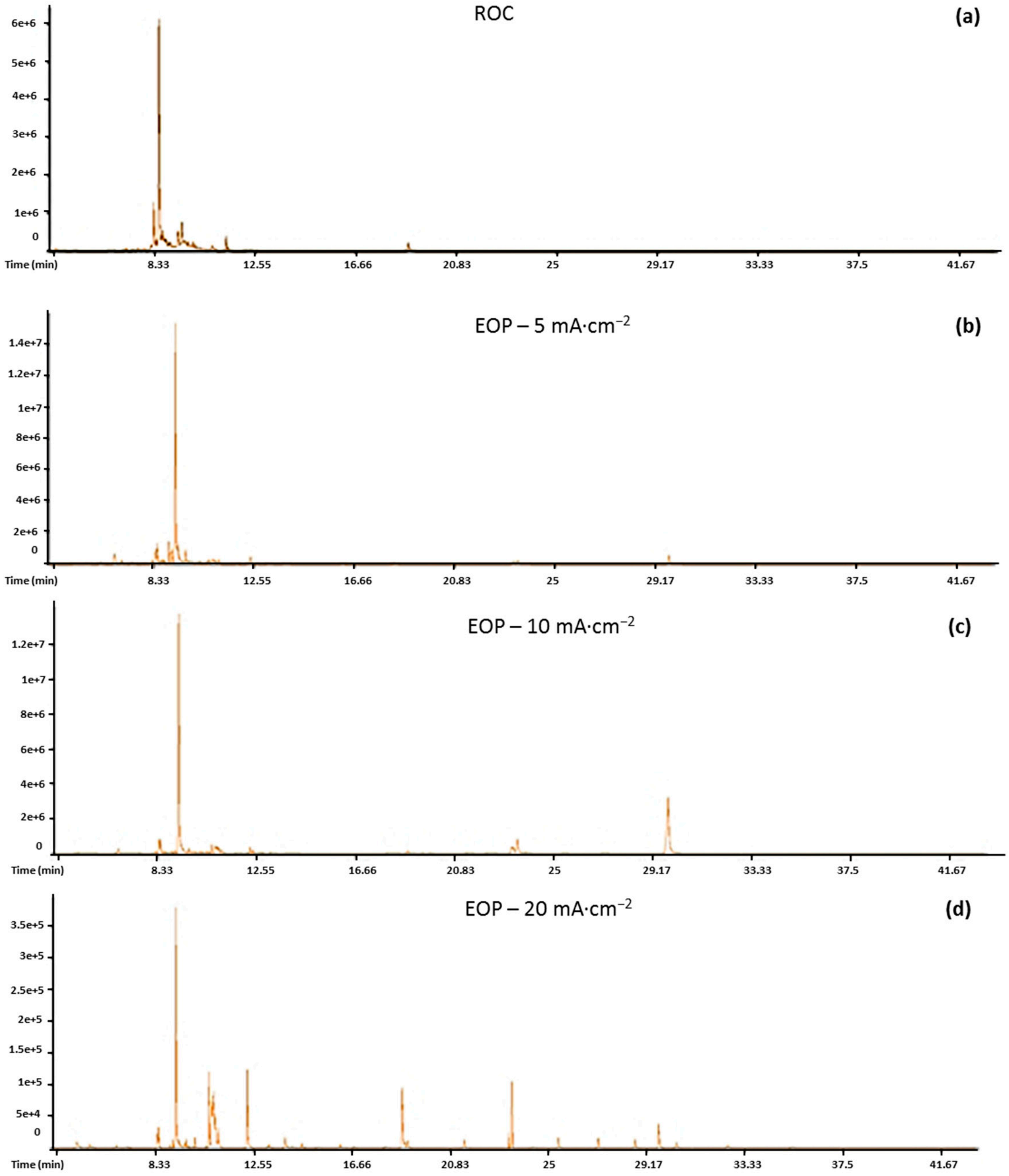


**Figure S2.** Chromatograms for **(a)** Reverse Osmosis Concentrate (ROC) before the application of EOP and after the EOP applying **(b)** 5 mA·cm$^{-2}$, **(c)** 20 mA·cm$^{-2}$ and **(d)** 30 mA·cm$^{-2}$.

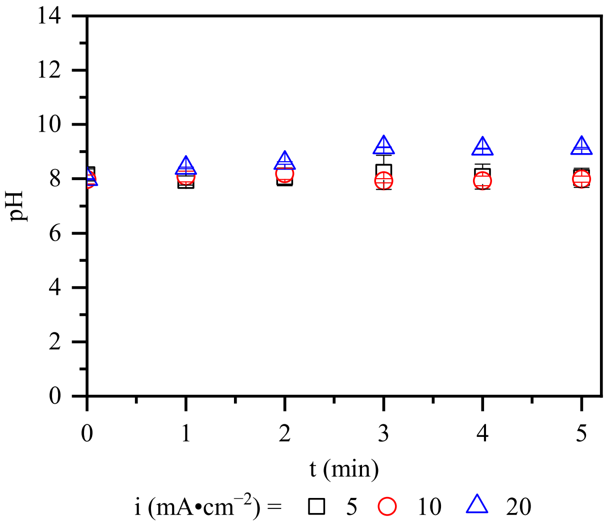


**Figure S3.** Influence of applied current densities on the pH.
